# Association of opioid receptor mu 1 (OPRM1) A118G polymorphism (rs1799971) with nicotine dependence

**DOI:** 10.18632/oncotarget.20939

**Published:** 2017-09-15

**Authors:** Xiangyi Kong, Hao Deng, Theodore Alston, Yanguo Kong, Jingping Wang

**Affiliations:** ^1^ Department of Neurosurgery, Peking Union Medical College Hospital, Chinese Academy of Medical Sciences, Dongcheng District, Beijing 100730, P. R. China; ^2^ Department of Anesthesia, Critical Care and Pain Medicine, Massachusetts General Hospital, Harvard Medical School, Harvard University, Boston, Massachusetts 02114-3117, United States of America

**Keywords:** OPRM1-A118G, rs1799971, polymorphism, nicotine dependence, meta-analysis

## Abstract

**Background and Object:**

Whether opioid-receptor mu 1 (OPRM1) A118G polymorphism (rs1799971) is associated with nicotine dependence is controversial. We analyzed the combined results from published studies of this possibility.

**Methods:**

Literature reviews were performed according to Preferred Reporting Items for Systematic Reviews and Meta-Analyses (PRISMA) guidelines. Web of Science, Chinese National Science Infrastructure (CNKI), PubMed, Embase and Google Scholar database searches using MeSH terms were conducted to find all relevant researches up to October 2016. Odds ratios (ORs) and their 95% confidence intervals (95% CIs) were calculated in allele, homozygote, heterozygote, dominant and recessive models. Ethnicity-specific subgroup meta-analysis, heterogeneity, sensitivity analysis and publication bias were considered.

**Results:**

Seven eligible studies with 3313 patients were included. The ORs in the five genetic models mentioned above were 1.000 (95% CI: 0.906, 1.104; p = 0.999), 1.032 (95% CI: 0.771, 1.381; p = 0.834), 0.963 (95% CI: 0.799, 1.162; p = 0.696), 1.006 (95% CI: 0.916, 1.104; p = 0.907), 0.967 (95% CI: 0.715, 1.309; p = 0.830), respectively. Only in dominant model is the association significant. Upon ethnicity-specific subgroup analysis, there is no statistical significance.

**Conclusion:**

OPRM1-A118G polymorphism (A>G) is not associated with nicotine dependence.

## INTRODUCTION

Nicotine dependence is one of the commonest behavioral disorders. It involves psychological and physical dependences on nicotine and loss of control of in spite of frequent undesirable complications [1]. Smoking is considered to be one of the independent causes of a series of severe illnesses such as stroke, pulmonary disease, cardiac-cerebral vascular disease, and cancer. In recent years, some studies implicate genetic factors in the susceptibility to smoking addiction [2, 3]. A number of candidate genes in the reinforcement and reward system may play vital roles in drug abuse, including that of nicotine dependence [4].

A significant neurotransmitter system relevant to nicotine-induced reward is the endogenous opioid system. Nicotine consumption can lead to increased endogenous opioids, especially β-endorphin. The binding of β-endorphin to μ-opioid receptors (genetic locus OPRM1) might reinforce nicotine dependence by increasing dopamine actions in reward centers [5, 6]. As suspected in the case of alcohol, genetic variations of OPRM1 might impact the risk of developing nicotine dependence. The exon 1 A118G (rs1799971) is in the OPRM1 coding area, leading to an Asn40Asp substitution of amino acids. Present studies of the possible association of nicotine dependence and OPRM1-A118G polymorphism evince mixed findings. Present studies are of small sample size, and we therefore performed a meta-analysis of the available case-controlled trials.

## RESULTS

### Search results and study features

**Figure 1:**
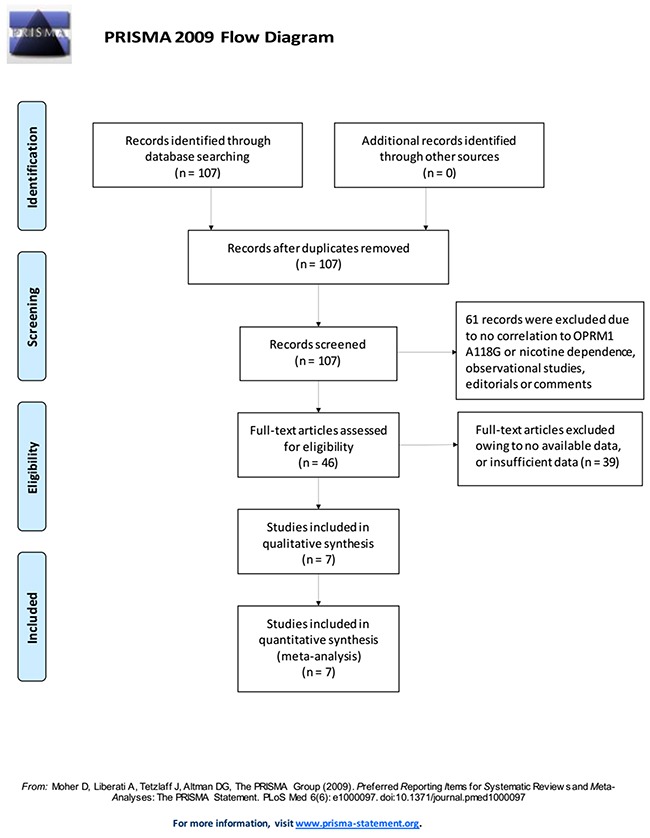
Literature search and selection of articles

Figure 1 outlines the literature search process. Based on the inclusion criteria set in Table 1, a total of seven articles involving 3313 patients were finally included [5, 7–11], among which four studies [5, 7, 10] involved predominantly white patients in the USA, Norway, and Spain (1596 cases in total). Three involved predominantly Asian patients [8, 9, 11] in mainland China [9, 11] and Taiwan [8] (1717 cases in total). All studies were reported in English. Nicotine dependence was defined by nicotine consumption and smoking history. In all included studies, distributions of the OPRM1-A118G polymorphism (A>G) in the controls were consistent with Hardy-Weinberg equilibrium. A variety of genotyping methods were applied including PCR-RFLP [8, 10], iPLEX/MALDI-TOF mass spectrometry [9], and TaqMan assay method [5, 7, 10, 11]. Genes were read from blood samples in all included studies. Controls were mainly matched in terms of age, and they were population-based in four studies [5, 7, 9, 10], hospital-based in two [10], and not so-specified in two [8, 11]. Literature methodological quality assessment scoring standard is shown in Table 2), and the explanations of some key statistical concepts are shown in Table 3. Study characteristics and quality assessment results are shown in Table 4.

**Table 1:** Inclusion criteria for study selection in this meta-analysis

Number	Inclusion criteria
1	Case-control studies.
2	The studies evaluated the associations between OPRM1 A118G polymorphism and nicotine dependence.
3	The studies included detailed genotyping data (total number of cases and controls, number of cases and controls with A/A, A/G, and G/G genotypes).
4	Studies focusing on human being.
**Number**	**Exclusion criteria**
1	The design of the experiments was not case-control.
2	The source of cases and controls, and other essential information were not provided.
3	The genotype distribution of the control population was not in accordance with the Hardy–Weinberg equilibrium (HWE).
4	Reviews and duplicated publications.

**Table 2:** Scale for methodological quality assessment

Criteria	Score
**1. Representativeness of cases**	
RA diagnosed according to acknowledged criteria.	2
Mentioned the diagnosed criteria but not specifically described.	1
Not Mentioned.	0
**2. Source of controls**	
Population or community based	3
Hospital-based RA-free controls	2
Healthy volunteers without total description	1
RA-free controls with related diseases	0.5
Not described	0
**3. Sample size**	
>300	2
200-300	1
<200	0
**4. Quality control of genotyping methods**	
Repetition of partial/total tested samples with a different method	2
Repetition of partial/total tested samples with the same method	1
Not described	0
**5. Hardy-Weinberg equilibrium (HWE)**	
Hardy-Weinberg equilibrium in control subjects	1
Hardy-Weinberg disequilibrium in control subjects	0

**Table 3:** Statistical methods used in this meta-analysis and their explanations

Statistic means	Goals and usages	Explanation
Labbe plot	To evaluate heterogeneity between the included studies	In Labbe figure, if the points basically present as a linear distribution, it can be taken as an evidence of homogeneity.
Cochran's Q test	To evaluate heterogeneity between the included studies	Cochran's Q test is an extension to the McNemar test for related samples that provides a method for testing for differences between three or more matched sets of frequencies or proportions. Heterogeneity was also considered significant if P < 0.05 using the Cochran's Q test.
I^2^ index test	To evaluate heterogeneity between the included studies	The I^2^ index measures the extent of true heterogeneity dividing the difference between the result of the Q test and its degrees of freedom (k – 1) by the Q value itself, and multiplied by 100. I^2^ values of 25%, 50% and 75% were used as evidence of low, moderate and high heterogeneity, respectively.
Sensitivity analysis	To examine the stability of the pooled results	A sensitivity analysis was performed using the one-at-a-time method, which involved omitting one study at a time and repeating the meta-analysis. If the omission of one study significantly changed the result, it implied that the result was sensitive to the studies included.
Funnel plot	Publication bias test	In the absence of publication bias, it assumes that studies with high precision will be plotted near the average, and studies with low precision will be spread evenly on both sides of the average, creating a roughly funnel-shaped distribution. Deviation from this shape can indicate publication bias.

**Table 4:** Characteristics of studies included in the meta-analysis

Author	Year	Country	Ethnicity	Disease type	Genotyping	Source of controls	Nicotine-dependence (n)				Controls (n)				P for HWE	Quality
							Total	AA	AG	GG	Total	AA	AG	GG		
Schinka	2002	USA	Caucasian	Nicotine -dependence	PCR-RFLP	Population-based	134	114	20	0	297	220	73	4	0.0000	8
Zhang	2006	China	Asian	Nicotine -dependence	Taqman	NA	443	343	90	10	238	187	46	5	0.313	8
Chen	2013	Taiwan, China	Asian	Nicotine -dependence	PCR-RFLP	NA	366	151	170	45	387	180	159	48	0.1678	6
Fang	2014	China	Asian	Nicotine -dependence	iPLEX/MALDI-TOF mass spectrometry	Population-based	137	64	62	11	146	72	58	16	0.4116	7
Hasvik	2014	Norway	Caucasian	Nicotine -dependence	Taqman	Population-based	43	34	9	0	75	61	13	1	0.7484	6
Frances	2015	Spain	Caucasian	Nicotine -dependence	Taqman	Population-based	175	118	54	3	588	408	166	14	0.549	8
Hirasawa	2015	USA	Caucasian	Nicotine -dependence	Taqman	Hospital-based	196	157	29	10	88	63	25	0	0.1204	7

### Meta-analysis results

**Figure 2:**
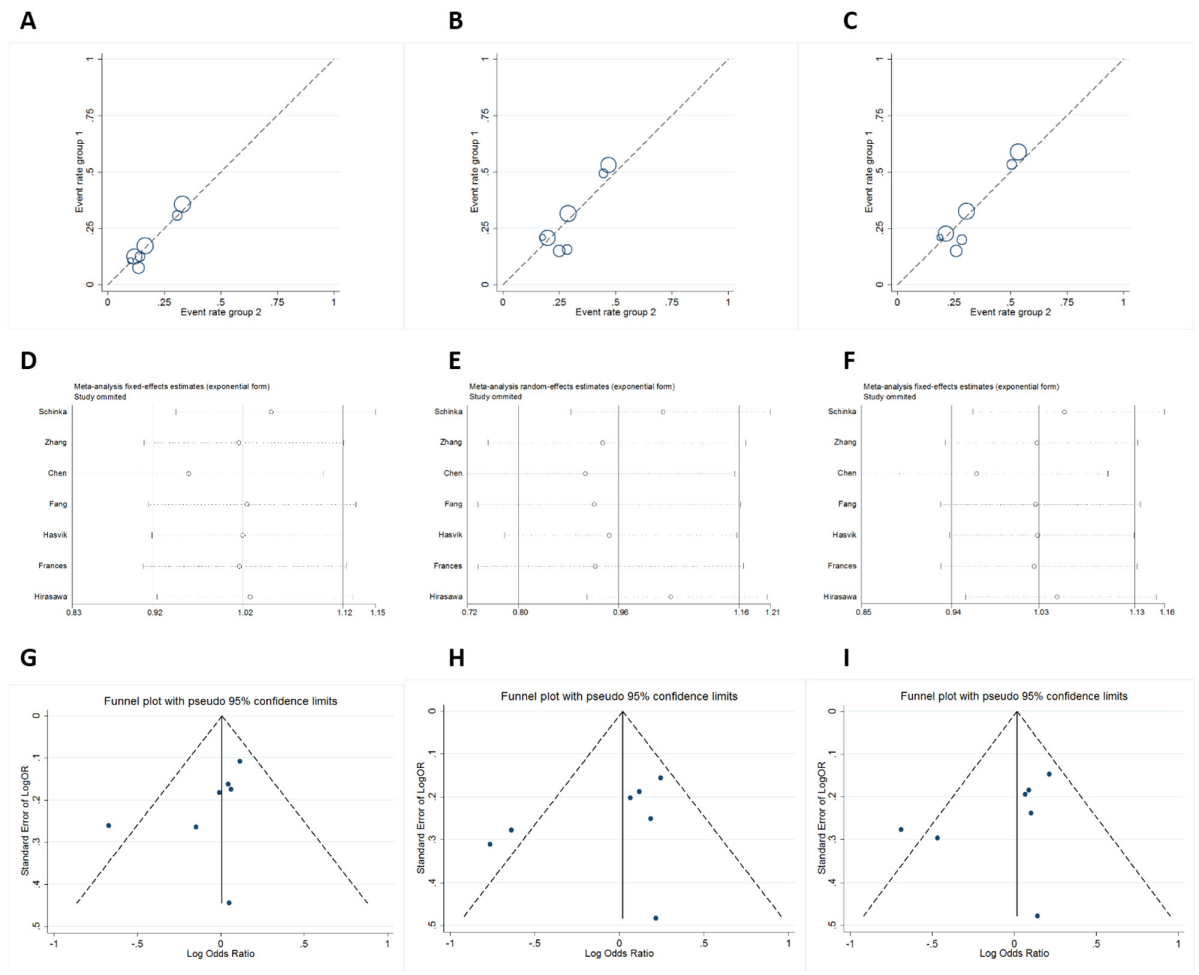
Labbe plots, sensitivity analysis plots and contour-enhanced funnel plots of the included studies focusing on the association between OPRM1-A118G Polymorphism and nicotine-dependence risk Labbe plots in allele model (**A**), heterozygote model (**B**), and dominant model (**C**). Sensitivity analysis in allele model (**D**), heterozygote model (**E**), and dominant model (**F**). Funnel plots in allele model (**G**), heterozygote model (**H**), and dominant model (**I**).

**Figure 3:**
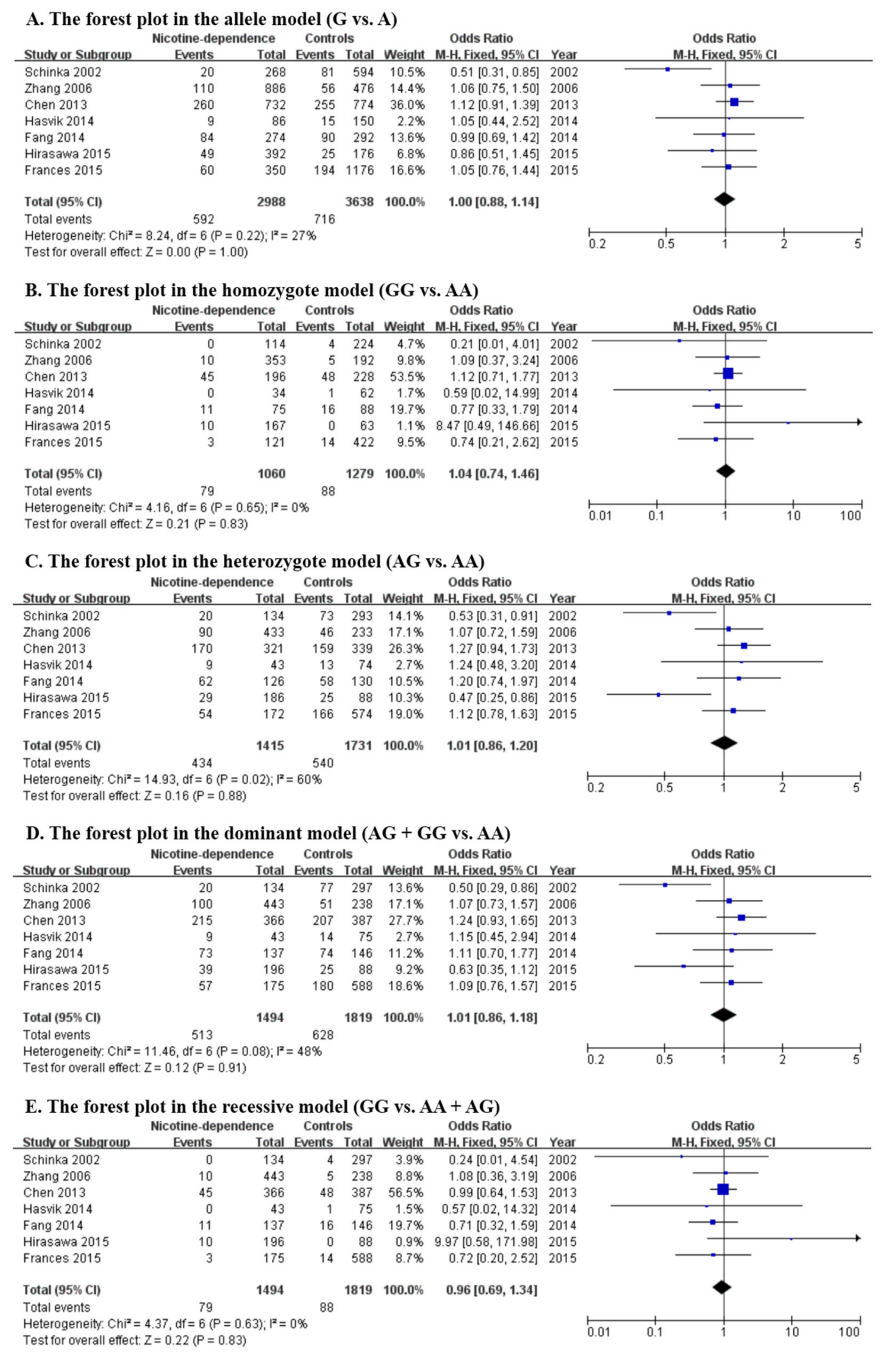
Forest plots (individual and pooled effects with 95% CI) regarding the association between OPRM1-A118G polymorphism and nicotine-dependence in allele model (**A**), homozygote model (**B**), heterozygote model (**C**), dominant model (**D**) and recessive model (**E**).

The main results including heterogeneity tests, effect models adopted accordingly, and the pooled OR with 95% CI and P value of this meta-analysis were shown in Table 5. The Labbe plots for allele model, heterozygote model and dominant model were shown in Figure 2A, 2B, 2C. In the overall level, the statistically correlation between OPRM1-A118G polymorphism and increased nicotine-dependence risks was not found in any of the five models (allele model: OR 1.000, 95% CI 0.906, 1.104; p = 0.999; Figure 3A; homozygote model: OR 1.032, 95% CI 0.771, 1.381; p = 0.834; Figure 3B; heterozygote model: OR 0.963, 95% CI 0.799, 1.162; p = 0.696; Figure 3-C; dominant model: OR 1.006, 95% CI 0.916, 1.104; p = 0.907; Figure 3D; recessive model: OR 0.967, 95% CI 0.715, 1.309; p = 0.830; Figure 3E).

**Table 5:** Results of meta-analysis for various genotype models

Genetic model			Heterogeneity test							Test of Association					Egger's test		
Name	Explanation	Ethnicity	Q value	d.f.	I-squared	Tau-squared	P Value	Heterogeneity	Effect model	Pooled OR	95% CI	Z value	P value	Statistical significance	P Value	95% CI	Publication bias
Allele model	G vs. A	Caucasian	5.70	3	47.3%	NA	0.127	No	Fixed	0.876	[0.719, 1.067]	1.32	0.187	No	-	-	-
		Asian	0.31	2	0.0%	NA	0.857	No	Fixed	1.056	[0.943, 1.183]	0.94	0.346	No	-	-	-
		Total	8.02	6	25.2%	NA	0.236	No	Fixed	1.000	[0.906, 1.104]	0.00	0.999	No	0.174	[-4.45, 1.05]	No
Homozygote model	GG vs. AA	Caucasian	3.54	3	15.3%	NA	0.315	No	Fixed	1.062	[0.439, 2.566]	0.13	0.895	No	-	-	-
		Asian	0.57	2	0.0%	NA	0.751	No	Fixed	1.027	[0.756, 1.395]	0.17	0.867	No	-	-	-
		Total	4.07	6	0.0%	NA	0.667	No	Fixed	1.032	[0.771, 1.381]	0.21	0.834	No	0.768	[-1.69, 1.32]	No
Heterozygote model	AG vs. AA	Caucasian	9.92	3	69.8%	0.1140	0.019	Yes	Random	0.797	[0.530, 1.197]	1.10	0.273	No	-	-	-
		Asian	0.16	2	0.0%	0.0000	0.923	No	Fixed	1.112	[0.984, 1.256]	1.70	0.089	No	-	-	-
		Total	14.66	6	59.1%	0.0332	0.023	Yes	Random	0.963	[0.799, 1.162]	0.39	0.696	No	0.228	[-7.24, 2.20]	No
Dominant model	AG+GG vs. AA	Caucasian	7.30	3	58.9%	NA	0.063	No	Fixed	0.862	[0.715, 1.039]	1.55	0.120	No	-	-	-
		Asian	0.15	2	0.0%	NA	0.928	No	Fixed	1.080	[0.971, 1.200]	1.42	0.157	No	-	-	-
		Total	11.02	6	45.5%	NA	0.088	No	Fixed	1.006	[0.916, 1.104]	0.12	0.907	No	0.195	[-6.22, 1.65]	No
Recessive model	GG vs. AA+AG	Caucasian	3.92	3	23.5%	NA	0.270	No	Fixed	1.133	[0.473, 2.711]	0.28	0.779	No	-	-	-
		Asian	0.58	2	0.0%	NA	0.748	No	Fixed	0.941	[0.682, 1.297]	0.37	0.710	No	-	-	-
		Total	4.29	6	0.0%	NA	0.638	No	Fixed	0.967	[0.715, 1.309]	0.21	0.830	No	0.984	[-1.53, 1.51]	No

Since ethnicity may have effect on this association, ethnicity-specific subgroup analysis was also performed. All ethnicities involved in these 7 articles can be divided into Caucasian group and Asian group. The subgroup results of heterogeneity tests and meta-analysis were also shown in Table 5 and Figure 3, from which, neither in Caucasian group nor in Asian group, the OPRM1-A118G polymorphism has correlation to nicotine-dependence. So at least for now, we cannot provide the evidence for the correlation based on the current circumstance.

### Sensitivity-analyses and publication-bias

The sensitivity-analyses suggested that the final OR was not influenced by removing each single literature (Figure 2D-2F). Funnel plots showed the overall symmetric distributions of the studies (Figure 2G-2I), indicating less likelihood of publication-bias. Meanwhile, according to Egger's test results, no significant publication bias was suggested for these included studies (p > 0.05, Table 5).

## DISCUSSION

In consideration of the significance of μ-opioid receptor systems in physiological mechanisms about the reward center, biologically, it is plausible that OPRM1 polymorphisms can modulate the risks of nicotine-dependence. Previously published reports demonstrated that OPRM1 A118A mRNAs were 1.5 to 2.5 folds more abundant than 118G mRNAs in cerebral homogenate, and 118G could lead to a 10 folds reduction at OPRM1-protein levels [12]. This indicated that the OPRM1-A118G was a functional allelic variant with damaging effect on both mRNA and protein production.

In the recent years, big data has established very close associations between OPRM1-A118G polymorphism and nicotine, alcohol, and opioid-dependence. Kapur et al. and Tan et al. found a positive correlation between the OPRM1-A118G polymorphism and heroin-dependences [13, 14]. Altered modulations of kinase A are considered to be responsible for the correlations [15]. Recently, Frances et al. found that OPRM1-A118G polymorphism (A>G) is closely related to alcohol/tobacco-dependence in Spanish people, and this association was affected by some environmental and genetic factors [5]. In females, Ray et al. found that there might be significant associations between nicotine reinforcements and the OPRM1-A118G haplotype [16]. Zhang and colleagues thought that it was some other markers combined within A1118G that were significantly associated with smoking initiation, instead of single OPRM1-A118G variant [11]. They found that another allele near the A118G locus serves as the actual risk factor [11]. Genome-wide association researches also showed that the OPRM1 gene is closely related to nicotine dependence [17].

A single study cannot confirm the correlation between OPRM1-A118G polymorphism and nicotine-dependence risks convincingly. This is particularly true for researches with relatively small sample-sizes. Given this, we pooled several databases to analyze the associations between nicotine-dependence and the OPRM1-A118G polymorphism. In our study, the statistically correlation between OPRM1-A118G polymorphism and increased nicotine-dependence risks was not detected in any of the five genetic models (OR 1.261, 95% CI 1.008, 1.578; p = 0.042). Also, different ethnicities might contribute to variable association findings. Thus, we also performed an ethnicity-based subgroup analysis. Similarly, no matter for Caucasian population or Asian population, the OPRM1-A118G polymorphism has no correlation to nicotine-dependence in all there five genetic models. Regarding the testing statistic, the integrated ORs were calculated. Generally, relative risk (RR) and OR are usually comparable in magnitude if the studied diseases are rare, like this case. However, using RR can sometimes magnify or overestimate risks, especially if the diseases are with higher incidence. We carefully reviewed our manuscript and related articles and we are happy to say in our meta-analysis, OR for study outcomes are comparable as RRs and these additional data is adding value to estimate a more accurate effect. In our meta-analysis, no publication bias was suggested according to the funnel-plot. We also conducted the Egger's test [18]. All p values were more than 0.05, indicating there was no significant publication bias.

There may be some limitations in our meta-analysis. Firstly, the number of the included literatures and the sample-size for each ethnicity were limited. Hence, type-II error couldn't be dismissed. Secondly, the effect of gene-environment interactions and gene-gene interactions was not emphasized because not all researches had this information, or even when they did, adjusted factors were reported differently. Thirdly, more accurate ORs should be adjusted by patient factors such as gender, age, living styles, medication consumption and other exposure factors. Fourth, only published articles were included, the unpublished and ongoing studies could convert our result.

## MATRIALS AND METHODS

### Publication search and selection criteria

Two authors searched Chinese National Knowledge Infrastructure (CNKI), Web of Science, PubMed, Embase and Google Scholar independently (cut-off date: 30 October 2016) to include case control researches about the correlation between the polymorphism of OPRM1-A118G (rs1799971) and nicotine-dependence risks. Search terms include “nicotine or tobacco or smoking” and “rs1799971 or A118G or OPRM1”. Relevant references were also searched to identify other potentially available researches. The inclusion-criteria and the exclusion-criteria are shown as Table 1.

### Data extraction

According to the inclusion criteria set in Table 1, two independent authors reviewed and extracted the needed data and information from the included articles. We collected the following information: author names, publication years, countries, ethnicities (Asian, Caucasian or others), genotyping ways, total numbers of respondents, numbers of controls and cases with OPRM1-A118G polymorphism, numbers of controls and cases with G/G, A/G and A/A genotype, control source (hospital-based or population-based), and P-value regarding Hardy-Weinberg equilibrium (HWE).

### Quality assessment

In accord with the methodological quality-assessment scale (see Table 2), that was adjusted from a previous publication, 2 authors estimated the qualities of the included literatures independently. Disagreement would be solved by discussion. In this methodological quality assessment scale, five items, including quality controls of genotyping ways, source of controls, sample sizes, cases representativeness and HWE were prudently checked. The quality scores range from 0 to 10, and high scores indicate good quality.

### Statistical analyses

This meta-analysis was in accordance with the PRISMA guidelines and checklists [19]. HWE in each study was firstly assessed, followed by the calculation of ORs with 95% CIs reflecting the correlation strength between OPRM1-A118G polymorphisms and the risks of nicotine-dependence. The integrated ORs were calculated and used for comparisons respectively in allele model (G vs. A), homozygote model (GG vs. AA), heterozygote model (AG vs. AA), dominant model (AG + GG vs. AA), and recessive model (GG vs. AA + AG). Ethnicity-specific subgroup (Caucasian and Asian) meta-analysis was also performed. The Labbe plot, I^2^ test and Cochran's Q-test (Table 3) were done for accessing the heterogeneities [20]. If no evidences of heterogeneities were suggested, the fixed-effects model would be chosen [21]. Otherwise, we chose the random-effects model. To access the stability, sensitivity-analyses are also necessary (explanation in Table 3) [22]. Using funnel plots and Egger linear regression tests (Table 3), potential publication biases were calculated. P < 0.05 indicates statistical significance.

## CONCLUSIONS

Opioid Receptor mu 1 (OPRM1) A118G Polymorphism (rs1799971) is not associated with nicotine dependence in white or Asian populations.

## Abbreviations

OPRM1: Opioid Receptor mu 1
CNKI: Chinese National Science Infrastructure
OR: odds ratio
CI: confidence interval
PCR-RFLP: Polymerase chain reaction restriction fragment length polymorphism
RR: relative risk
